# Antiulcer Effect of Genus *Symphytum* L. and *Portulaca oleracea* L. as Investigated on Experimental Animals

**DOI:** 10.1155/2024/9208110

**Published:** 2024-09-17

**Authors:** El-Sayed H. Bakr, Amal Al-Ghamdi, Reham Al-Amri, Muna Al-Otaibi, Nada Al-Saad, Ghala Al-Matrafi, Ziad T. Kishmira, Firas Azzeh, Areej A. Almuraee

**Affiliations:** ^1^ Department of Clinical Nutrition Faculty of Applied Medical Sciences Umm Al-Qura University, Makkah, Saudi Arabia; ^2^ Kafaat Medical Center, Makkah, Saudi Arabia

## Abstract

Peptic ulcer disease (PUD) occurs when open sores, or ulcers, form in the stomach or first part of the small intestine caused by bacterial infection (*H. pylori*) and/or nonsteroidal anti-inflammatory drug (NSAID) use. This study was conducted to evaluate the antiulcer effect of some plants including genus *Symphytum* L., and *Portulaca oleracea* L. on aspirin-induced acute gastric ulcer in rats. Sixteen male albino rats (200–210 g b.wt. each) were divided into 4 groups, 4 rats each, one of them left as the control −ve group while the other 3 groups orally administered with aspirin at a dose of 200 mg/kg b.wt., for gastric ulcer induction, one of them left as control +ve and the rest 2 groups were orally administered with genus *Symphytum* L. and *Portulaca oleracea* L. at a dose of 100 mg/kg b.wt., each. for seven consecutive days. Body weight gain (BWG), the length of gastric ulcer, the volume of gastric juice, the total acidity of gastric juice, and blood sample were assessed. The results showed that orally administered with genus *Symphytum* L. and *Portulaca oleracea* L. significantly reduced the length of gastric ulcer, gastric juice volume, and total acidity of gastric juice, in addition to decreasing total cholesterol (TC), triglyceride (TG), aspartate aminotransferase (AST), alanine aminotransferase (ALT), alkaline phosphatase (ALP), RBC, WBC, HGB, and PLT. No significant changes were observed in the pH of gastric juice among treated groups. Moreover, in comparison to *Portulaca oleracea* L., genus *Symphytum* L. showed preferable results. Accordingly, genus *Symphytum* L. and *Portulaca oleracea* L. could be used as plants as curative agents against gastric ulcer in experimental rats.

## 1. Introduction

Peptic ulcer disease occurs when open sores, or ulcers, form in the stomach or first part of the small intestine. Many cases of peptic ulcer disease arise due to bacterial erosion of the protective lining of the digestive system. In addition, habitual consumption of pain relievers increases the risk of peptic ulcer development. Peptic ulcer disease is a condition in which painful sores or ulcers develop in the lining of the stomach or the first part of the small intestine (the duodenum). Normally, a thick layer of mucus protects the stomach lining from the effect of its digestive juices. But many things can reduce this protective layer, allowing stomach acid to damage the tissue. There are a lot of medicinal plants that could be used for preventing and treating the complications of peptic ulcer [1].

It is believed that NSAIDs and aspirin may cause harm to the mucosa of the stomach and duodenum by inhibiting the synthesis of mucosal prostaglandin. This is considered a significant mechanism of gastrointestinal mucosal injury [1]. Selective cyclooxygenase (COX)-2 inhibitors produce less gastric damage than conventional nonsteroidal anti-inflammatory drugs (NSAIDs), suggesting that NSAIDs cause damage by inhibiting COX-1 leading to limiting mucus and bicarbonate secretion, slowing mucosal blood flow, impaired the blood platelet aggregation, and altered the structure of microvascular [2, 3].

Genus *Symphytum* L. (*Symphytum officinale*) is a plant that can be found commonly throughout Europe and parts of Asia and has also naturalized in North America where it has spread quickly. Native Americans have also recognized its healing properties and have used it in their treatments. Moreover, genus *Symphytum* L. has been used in veterinary medicine [4]. However, genus *Symphytum* L. formulations have been applied topically to treat episiotomy discomfort, cracked, sore nipples, fractured bones, lung congestion, tendon damage, gastrointestinal ulcerations, wound healing, and/or joint inflammation [5, 6]. Allantoin as an active ingredient of genus *Symphytum* L. has been connected to genus *Symphytum* L.'s capacity to heal wounds. Also, it is used for skin protection; therefore, it has been included as an ingredient of several cosmetic products and antiulcer drugs. Previously, it was stated that the level of allantoin in genus *Symphytum* L. leaves was around 1 mg/g [5].

*Portulaca oleracea* L., a member of the Portulacaceae family, is an annual herb that thrives in warm climates. It is commonly known as khurfa in Arabic and common purslane in English, and it can be found in various tropical and subtropical regions worldwide, including many parts of the United States. This plant is often consumed as a vegetable and utilized for medicinal purposes. Throughout history, purslane has been used to treat a variety of ailments, including skin diseases, fever, dysentery, diarrhoea, bleeding piles, and kidney, liver, and spleen diseases [7]. Due to its diverse phytoconstituents, purslane has shown to possess many pharmacological properties, such as hepatoprotective, neuroprotective, anti-inflammatory, antimicrobial, antidiabetic, antioxidant, anticancer, antihypertensive, and antiulcerogenic actions [8]. Accordingly, this study was conducted to evaluate the antiulcer effect of some promising medicine plants including genus *Symphytum* L. and *Portulaca oleracea* L. on aspirin-induced acute gastric ulcers in rats.

## 2. Materials and Methods

### 2.1. Aspirin and Medical Herbs

Aspirin (aspegic acid) was purchased from Arabian Chemical Co. (Jeddah, KSA), and the medicinal herb *Portulaca oleracea* L. was purchased from the local market in Holy Makkah, genus *Symphytum* L. from Amazon by online purchasing.

### 2.2. Rats

Sixteen Sprague–Dawley male albino rats with an average weight of 200 ± 10 g were obtained from the Department of Medical Biochemistry, Faculty of Medicine, Umm Al-Qura University, Holy Makkah, Saudi Arabia.

### 2.3. Methods

#### 2.3.1. Plant Extraction

The first plant, genus *Symphytum* L., dried leaves, was extracted by the infusion process in water at 70°C water for 15 minutes within a glass container before being filtered through paper [9]. The second plant, *Portulaca oleracea* L., was extracted by dissolving in ethanol for 24 hours, then the precipitate active ingredient was separated by a rotary evaporator [10].

#### 2.3.2. Preparing Rats for the Experiment

The experimental rats were housed in a clean and sterile polyvinyl cage in a room maintained at 22–24 C and 12 h light/dark cycle. The animals were kept on water ad libitum and basal diet for seven days for acclimatization before the beginning of the experiment. They were administered orally with extracts from the plants genus *Symphytum* L. and *Portulaca oleracea* L. for 7 consecutive days.

#### 2.3.3. Basal Diet

Reeves et al. [11] show that the basal diet was prepared of sucrose (10%), choline chloride (2%), casein (20%), salt mixture (4.7%), vitamin mixture (1%), corn oil (3.5%), and cellulose (5%). The remaining component will be corn starch (up to 100%).

#### 2.3.4. Peptic Ulcer Induction

Peptic ulceration was induced by orally administrated with 200 mg/kg b.wt in the animals according to the procedure described by Agrawal et al. [12]. The administration of treatment to animals began 1 day following ulcer induction.

#### 2.3.5. Experimental Design

Rats were divided randomly into four groups as follows:

  Group 1: fed on basal diet only as a control negative (C −ve) group for 7 consecutive days
  Group 2: fed on basal diet only and oral injection with aspirin at a dose of 200 mg/kg b.wt as a control positive (C +ve) group for 7 consecutive days
  Group 3: fed on basal diet with oral injection with aspirin at a dose of 200 mg/kg b.wt. and genus *Symphytum* L. at a dose of 100 mg/kg b.wt. for 7 consecutive days, according to the dose carried out by (Da Silva et al.) [13]
  Group 4: fed on basal diet and oral injection with aspirin at a dose of 200 mg/kg b.wt. with *Portulaca Oleracea* L. at a dose of 100 mg/kg b.wt. for 7 consecutive days, according to the dose carried out by Kumara et al. [14]

All the animals were fasted for 12 hours before being sacrificed on the next day.

#### 2.3.6. Measurement of the Volume of Gastric Juice

At the end of the experimental period, all rats were fasted for 12 hours, during which they were deprived of food but had ad libitum access to tap water. The rats were anesthetized with diethyl ether, followed by a laparotomy. Subsequently, the stomach was excised. The stomachs of each rat were weighed, wrapped around the pyloric and cardiac sphincter apertures, and injected with 3 ml of distilled water to collect gastric juice. The stomachs were then centrifuged at 1500 rpm for 15 minutes. A graduated cylinder was used to measure and convert the amount of gastric juice into millilitres (mL).

#### 2.3.7. Measurement of the Length of Gastric Ulcer

The stomachs of every rat were longitudinally opened, cleaned in saline, and then examined under a dissecting microscope. Following the procedure outlined by Akhtar and Ahmad [15], the length of the stomach ulcer was measured for each group and expressed as mean + SE.

#### 2.3.8. Determination of the Total Acidity and pH of Gastric Juice

By titrating 1 ml of gastric juice in 10 ml of distilled water with 0.01N NaOH and two drops of phenolphthalein as an indicator, total acidity was ascertained. Percentages were used to express the data. A pH meter was used to measure the pH level.

#### 2.3.9. Blood Sampling

Animals from each group were sacrificed at the end of the experiment, and the blood was collected in a clean, dry centrifuge tube. The blood was then centrifuged to separate the serum by centrifuging at 4000 RPM for 10 minutes at room temperature, followed by keeping the serum in a plastic vial (well stoppered) until analysis.

#### 2.3.10. Blood Analysis

Total cholesterol (TC), triglyceride (TG), aspartate aminotransferase (AST), alanine aminotransferase (ALT), and alkaline phosphatase (ALP), red blood cells (RBC), white blood cells (WBC), haemoglobin (HGB), and platelets (PLT) were determined.

### 2.4. Ethical Approval

The University of Umm Al-Qura approved and evaluated this study before any data were collected (number:HAPO-02-K-012-2030-11-1877). Ensure that all areas of animal experimentation are done in line with the highest ethical standards.

### 2.5. Statistical Analysis

Statistical analysis will be by one-way analysis of variance (ANOVA) to test differences in means of variables between groups, and *p* < 0.05 will be considered statistically significant. All data will be analysed by using the IBM Statistical Package for Social Sciences (SPSS) for Windows, version 28.0 (Armonk, N.Y.: IBM Corp).

## 3. Results and Discussion

### 3.1. Chemical Composition of Medicinal Plants

Genus *Symphytum* L. and *Portulaca oleracea* L., are two botanical species that have been utilized for medicinal purposes for centuries. Genus *Symphytum* L. is rich in several constituents, including allantoin (0.6–0.8%), tannins (4–6%), furcate (15–30%_, silicic acid (4%) as reported by Stickel and Seitz [16], vitamin A (0.63%), vitamin B12 (leaf), and vitamin E as for Thoresen and Eve [17]. On the other hand, *Portulaca oleracea* L. contains various essential compounds, such as thiamine (0.047 mg/100 g), niacin (0.480 mg/100 g), riboflavin (0.112 mg/100 g), vitamin C (21 mg/100 g), calcium (65 mg/100 g) as stated by Chugh et al. [8], zinc (0.17 mg/100 g), and iron (1.99 mg/100 g) according to Viana et al., [18]. In addition, *Portulaca Oleracea* L. is a rich source of kaempferol (11 mg/kg) indicated by Ozcan and Yaman [19] and apigenin (6.17 *μ*g) reported by Junjun Ai et al. [20].

### 3.2. Effects of Plant Extracts on Body Weight Gain in Rats Inflicted with Gastric Ulcer

The effects of genus *Symphytum* L. and *Portulaca oleracea* L. at a dose of 100 mg/kg b.wt., each on body weight gain in rats inflicted with gastric ulcer in rats inflicted with gastric ulcer is listed in Table 1.

It could be observed for control positive rats that body weight gain was 10.00 ± 0.89 g/7 consecutive days, compared to 27.50 ± 0.09 g/7 consecutive days for control negative (*p* < 0.05). These results denote that there was a significant decrease in body weight gain in rats inflicted with gastric ulcers compared to normal rats. All rats were orally administered with genus *Symphytum* L. and *Portulaca Oleracea* L. at a dose of 100 mg/kg b.wt. Rats orally administered with genus *Symphytum* L. showed a significant increase in BWG as compared to control positive which were 10.00 ± 0.89, 16.60 ± 0.52, and 10.90 ± 0.29 g/7 day, while rats orally administered with *Portulaca Oleracea* L. did not reflect improvement in BWG which was 10.90 ± 0.29 g/7 days compared to all groups.

### 3.3. Effects of Plant Extracts on the Length of Gastric Ulcer in Rats Inflicted with Gastric Ulcer

The effects of genus *Symphytum* L. and *Portulaca Oleracea* L. at a dose of 100 mg/kg. b.wt., each on the length of gastric ulcer in rats inflicted with gastric ulcer are listed in Table 2.

It could be observed that the length of gastric ulcer in group No.2 (control +ve group) was 2.03 ± 0.09 mm., compared to group No. 1 (control −ve group) which was (0.00). This reflects a significant increase in gastric ulcer length in the group No.2 (control +ve group) as compared to the group no. 1 (control −ve group). All rats orally administered with genus *Symphytum* L. showed significant decrease in gastric ulcer length as compared to group no. 2 (control +ve group) which were 0.91 ± 0.02 mm. As for group no.4 (*Portulaca oleracea* L.), 1.21 ± 0.05 mm showed a significant decrease in gastric ulcer length as compared to group no.1 (control −ve group). Moreover, in comparison to other groups, group no. 3 (genus *Symphytum* L.) had the best results.

### 3.4. Effects of Plant Extracts on the Volume of Gastric Juice in Rats Inflicted with Gastric Ulcer

The effects of genus *Symphytum* L. and *Portulaca oleracea* L. at a dose of 100 mg/kg b.wt. each on the volume of gastric juice in rats induced by gastric ulcer is listed in Table 3.

It could be observed for group no. 2 (control +ve group) that the volume of gastric juice was 2.30 ± 0.08 mL compared to 1.33 ± 0.03 mL for group no. 1 (control −ve group) (*p* < 0.05). These results were significant. All rats orally administered with genus *Symphytum* L. and *Portulaca Oleracea* L.*, at* a dose of 100 mg/kg b.wt., each showed a significant decrease in volume of gastric juice with values of 1.50 ± 0.04, 1.70 ± 0.07, and 2.30 ± 0.08 2.30 ± 0.08 mL, respectively. For group no. 3 (genus *Symphytum* L.) showed a significant decrease in the volume of gastric juice when compared with group no. 4 (*Portulaca oleracea* L.) with values of 1.50 ± 0.04 and1.70 ± 0.07 mL, respectively. In addition, group no. 3 (*genus Symphytum* L.) was the best group in comparison to the other group.

### 3.5. Effects of Plant Extracts on pH of Gastric Juice in Rats Inflicted with Gastric Ulcer

The effects of genus *Symphytum* L. and *Portulaca oleracea* L. at a dose of 100 mg/kg b.wt. each on the pH of gastric juice in rats induced by gastric ulcer is listed in Table 4.

As listed in Table 4, the result of this study found a significant change in pH of gastric juice for rats in group no. 2 (control +ve group], which orally administered a dose of 200 mg/kg b.wt. of aspirin compared with group no. 1 (control −ve group) (5.60 ± 0.09 ab and 4.70 ± 0.04 d, respectively). All rats orally administered with genus *Symphytum* L. and *Portulaca oleracea* L. at a dose of 100 mg/kg b.wt., each showed insignificant changes in the pH of gastric juice when compared to group no. 2 (control +ve group).

### 3.6. Effects of Plant Extracts on the Total Acidity of Gastric Juice in Rats Inflicted with Gastric Ulcer

The effects of genus *Symphytum* L. and *Portulaca oleracea* L. at a dose of 100 mg/kg b.wt. each on the total acidity of gastric juice in rats induced by gastric ulcer are listed in Table 5.

The results indicate a significant increase in total acidity in gastric juice in rats in the control +ve group compared to the (control −ve group]), with values of 1.00 × 10^−1^ ± 0.005. As compared to the (control +ve group), all groups of ulcerated rats treated with genus *Symphytum* L. and *Portulaca oleracea* L. showed a significant decrease in the percentage of total acidity. However, in group 3, the genus *Symphytum* L. dose showed the highest reduction in the percentage of total acidity in rats compared to (control +ve group), 3.50 × 10^−2^ ± 0.003 and 1.00 × 10^−1^ ± 0.005, respectively. Furthermore, the dose of *Portulaca oleracea* L. showed the lowest reduction in total acidity of gastric juice compared with (control +ve group) of 5.00 × 10^−2^ ± 0.002 and 1.00 × 10^−1^ ± 0.005, respectively.

### 3.7. Effects of Plant Extracts on TG and TC of Gastric Juice in Rats Inflicted with Gastric Ulcer

The effects of genus *Symphytum* L. and *Portulaca oleracea* L. at a dose of 100 mg/kg. b.wt. each on TG and TC in rats induced by gastric ulcer are listed in Table 6.

As cleared from Table 6 for group no. 2 (control +ve group), the results for TG and TC were 98.92 ± 2.62 and 130.30 ± 3.02 mg/dl for 7 consecutive days, Compared to 80.24 ± 1.03, 78.24 ± 1.19 1 mg/dl group no. 1 (control −ve group) for 7 consecutive days (*p* < 0.05). All rats orally administrated with genus *Symphytum* L. at a dose of 100 mg/kg b.wt. with a value of TG and TC was 80.24 ± 1.03 c and 78.24 ± 1.19 c mg/dl, respectively, showed a significant result when compared with group no. 2 (control +ve group). For orally administered with *Portulaca oleracea* L. at a dose of 100 mg/kg b.wt., values of (TG and TC) 95.15 ± 0.09 b and 103.31 ± 2.37 mg/dl b, respectively, showed a significant result when compared with group no. 2 (control +ve group). For group no. 3 (genus *Symphytum* L.), values of (TG and TC) 80.24 ± 1.03 c and 78.24 ± 1.19 c mg/dl, respectively, showed significant results when compared with group no. 4 (*Portulaca oleracea* L.) with values of (TG and TC) 95.15 ± 0.09 b and 103.31 ± 2.37 b mg/dl, respectively. We observed group no. 3 (genus *Symphytum* L.) had a better significant effect on TG and TC more than group no. 4 (*Portulaca oleracea* L.).

### 3.8. Effects of Plant Extracts on AST, ALT, and ALP of Gastric Juice in Rats Inflicted with Gastric Ulcer

The effects of genus *Symphytum* L. and *Portulaca oleracea* L at a dose of 100 mg/kg b.wt each on AST, ALT, and ALP in rats induced by gastric ulcer are listed in Table 7.

It could be observed for group no. 2 (control +ve group) that AST, ALT, and ALP were 130.13 ± 3.07, 116.40 ± 2.30, and 292.90 ± 5.16 U/L for 7 consecutive days, compared to 45.92 ± 2.01, 41.25 ± 1.17, and 146.40 ± 4.07 U/L for 7 consecutive days for group no. 1 (control −ve group) (*p* < 0.05). These results denote that there was a significant increase in AST, ALT, and ALP in rats inflicted with gastric ulcers compared to normal rats. All rats were orally administered with genus *Symphytum* L. and *Portulaca oleracea* L. at a dose of 100 mg/kg b.wt. showed a significant decrease in AST, ALT, and ALP as compared to control-positive rats. Rats orally administered with genus *Symphytum* L. reflected the highest significant decrease in AST, ALT, and ALP compared to *Portulaca oleracea* L.

### 3.9. Effects of Plant Extracts on RBC, WBC, HGB, and PLT of Gastric Juice in Rats Inflicted with Gastric Ulcer

The effects of genus *Symphytum* L., and *Portulaca oleracea* L. at a dose of 100 mg/kg b.wt. each on RBC, WBC, HGB, and PLT in rats induced by gastric ulcer are listed in Table 8.

It could be observed in group no. 2 (control +ve group), and the blood test results for RBCs, WBCs, HGB, and PLTs were significantly different from those of group no. 1 (control −ve group) (*p* < 0.05). All rats orally administered with genus *Symphytum* L. at a dose of 100 mg/kg b.wt., and the blood test results for RBCs, WBCs, HGB, and PLTs showed a significant result when compared to group no. 2 (control +ve group). For rats orally administered with *Portulaca oleracea* L. *at* a dose of 100 mg/kg b.wt., the blood test results for RBCs, WBCs, and PLTs showed a significant result, except for HGB which showed an insignificant result when compared to group no. 2 (control +ve group). Moreover, for group no. 3 (genus *Symphytum* L.), the blood test results for WBCs, HGB, and PLTs showed significant results when compared with group no. 4 (*Portulaca oleracea* L.),except for red blood cells (RBCs), showed an insignificant result. In addition, group no. 3 (genus *Symphytum* L.) was the best group in comparison to the other group.

## 4. Discussion

This study investigated the effects of genus *Symphytum* L. and *Portulaca oleracea* L. on gastric ulcer and lipid profile in rats. The results showed that *both* genus *Symphytum* L., and *Portulaca oleracea* L. extract significantly reduced the gastric ulcer index and ulcer length and increased gastric mucus production and gastric mucosal thickness. In addition, both extract significantly reduced serum total cholesterol, triglyceride, and LDL cholesterol levels.

Our findings in Table 1 are consistent with Ezejindu et al. [21] who reported a significant increase in body weight in rat-administered genus *Symphytum* L. at a low dose (0.4 ml/28 days) compared to the control group.

However, our findings in contrast to some of the previous findings [22], mentioning the results of the group treated with *Portulaca oleracea* L., were shown a significantly lower rate of weight loss (AA2) by inhibiting the oxidative stress response through the MDA, NO, and SOD activities, reducing the mRNA expressions of proinflammatory cytokines (TNF-*α*, IL-1*β*, and IL-6) and the protein expressions of TNF-*α* and NF-kB p65, which led to increasing the colon length, decreasing body weight loss, and the disease activity index score compared to the positive control, which may be attributed to antioxidant properties of *Portulaca oleracea*'*s* components, including gallotannins, omega-3 fatty acids, ascorbic acid, tocopherols, kaempferol, quercetin, and apigenin [23].

Florentino et al. [24] agree with our results in Table 2 indicating that the extract of the genus *Symphytum* L. has an anti-inflammatory effect, with allantoin functioning as the active ingredient, which is an alternative to drugs, especially for stomach diseases, including stomach ulcers. Moreover, Zhou et al. [23] mentioned that *Portulaca oleracea* L. has an anti-inflammatory effect by inhibition of tumor necrosis factor (TNF-) *α*-induced production of intracellular reactive oxygen species (ROS) and overexpression of intercellular adhesion molecule (ICAM-)1, vascular cell adhesion molecule (VCAM)-1, and E-selectin in human umbilical vein endothelial cells (HUVECs) in a dose-dependent manner. This extract also suppresses the translocation of nuclear factor *κ*B (NF-*κ*B) p65 to the nucleus, TNF-*α*-induced NF-*κ*B binding, and the degradation of inhibitor molecule (I*κ*B)-*α*. Furthermore, an inhibition in the adhesion of HL-60 cells to TNF-*α*-induced HUVECs and TNF-*α*-induced mRNA expression of interleukin (IL).

Experimental studies have shown that ulcer models are highly sensitive because they generate an increase in aggressive factors and a decrease in defensive parameters [13]. *Portulaca oleracea* L. is a herb that has been used traditionally to treat a variety of diseases, including ulcers, due to its phytochemical composition, which includes saponins, omega-3 fatty acids, flavonoids, phenolic acids, and antimicrobial properties [7].

Genus *Symphytum* L. contains allantoin, a compound with antiulcer and antisecretory properties. As shown in Tables 3 and 5, our data suggest that allantoin can reduce gastric acid secretion systemically, which is in line with the findings of Falcão et al. Our data are also consistent with the authors in [21], who reported that allantoin suppressed gastric acid secretion in a pylorus ligature model, demonstrating its clinical antisecretory properties. It is likely that the increase in PGE2 levels is due to allantoin's antisecretory and cytoprotective effects. In addition, allantoin reduces vascular permeability and MPO activity, which are key parameters of gastrointestinal acid secretion. According to other study, there is another component in genus *Symphytum* L. which contribute to the antisecretory properties, which is rosmarinic acid that can lead to a significantly diminished gastric secretion volume [25]. The reduction in gastric juice may be due to a decreasing thyroid hormone level, which has reduced the number of parietal cells that are secreting stomach juices. It is also possible that thyroid hormone effects can be influenced by the size or metabolic function of parietal cells [26]. Since NSAID-induced COX-1 inhibition in the gastrointestinal tract reduces prostaglandin secretion [27], allantoin has been previously reported to increase the activity of the COX production pathway. Therefore, increased COX-1 and COX-2 synthesis may lead to increased PGE2 secretion in gastric tissues [28]. Furthermore, allantoin retains PGE2 content as it causes the rat gastric corpus to produce more prostaglandin-like substances, which indicate higher outputs of PGF2 and 6-keto-PG. Prostaglandins can protect the gastric mucosa by suppressing acid secretion, stimulating mucus and bicarbonate secretion, and altering mucosal blood flow [29]. Several components may potentially contribute to the antiulcer activity of genus *Symphytum* L., particularly in relation to the elevation of PGE levels. According to certain studies, the presence of ascorbic acid in genus *Symphytum* L. can lead to a notable increase in the production of PGE2 and PGF2 in dormant cells [30]. In addition, other studies suggested that prior exposure to varying concentrations of tannin can result in a marked reduction in the ulcer index, possibly due to an enhanced synthesis of prostaglandins [31].

As listed in Table 4, our findings disagree with other studies that claim ethanolic extracts of *Portulaca oleracea* L. that demonstrated an elevation of the pH of gastric juice in rats with pylorus ligation [22]. Gastric acid and pH are crucial factors in ulceration [32]. In a different study, pepsin and acid discharges were significantly reduced, indicating that the pH of gastric juice had increased [14]. Another study concluded that when rats received oral injections of allantoin, the pH has increased [13]. Our data surmised that the discrepancy between our study and others may be related to the length of time the rats with ulcers were treated.

The correlation between aspirin-induced peptic ulcers and lipid profiles unveils a complex relationship involving inflammation and elevated levels of LDL cholesterol, which can disrupt the integrity of the gastric mucosa and increase oxidative stress [33]. A study highlighted the antihyperlipidemic effects of *Portulaca oleracea* L., resulting in a significant reduction in total lipid and total cholesterol levels, in line with our own findings presented in Table 6. In addition, *Portulaca oleracea* L. exhibited potential in normalizing levels of total lipids, triglycerides (TG), and total cholesterol (TC) through the presence of two active components, apigenin and kaempferol, demonstrating potent scavenging abilities against free radicals, such as reactive oxygen species (ROS), thereby promoting the healing of the gastric mucosa [8]. Furthermore, mechanisms such as enhancing lipase enzyme activity or increasing TG excretion in stool were identified to significantly reduce TG levels [34, 35]. Another study indicated that phenolics' ability to decrease total cholesterol (TC) can be attributed to the rapid degradation of LDL-c through its hepatic receptors before eventual excretion as bile acids [36, 37]. These combined effects indirectly contributed to the normalization of lipid profiles, ultimately enhancing the healing of the gastric mucosa. Some biomolecules in genus *Symphytum* L., renowned for their hypoglycemic properties, are likely due to the presence of polysaccharides. Polysaccharides contain hydroxyl groups (-OH) capable of donating hydrogen atoms to neutralize free radicals, influencing lipid metabolism and promoting healing of the gastric mucosa [38]. However, further investigation into the underlying mechanisms is warranted.

The findings in Table 7 demonstrate that administering *Symphytum officinale* at 100 mg/kg body weight significantly decreased AST, ALT, and ALP levels in rats with gastric ulcers. This contrasts with the study by Ezejindu et al. [39], who reported increased liver enzyme levels at higher doses of *Symphytum officinale*. The discrepancies may be due to differences in dosage, extract preparation, and experimental conditions. While Ezejindu et al. suggest potential hepatotoxicity at higher doses, our results indicate potential hepatoprotective effects at a lower dose, particularly in the context of gastric ulcers. This underscores the need for further research to understand the varying effects of *Symphytum officinale*. In addition, our result agreed with that of Liu et al. [40]. Shi et al. [41], who found ethanol extract from *Portulaca oleracea* L., could attenuate acetaminophen-induced liver injury and carbon tetrachloride-induced liver injury in mice.

In Table 8, the CBC test results of rats treated with genus *Symphytum* L. and *Portulaca oleracea* L. at 100 mg/kg body weight were improved, especially in group 3 (genus *Symphytum* L.). This may be attributed to the anti-inflammatory properties of these plants. Furthermore, allantoin, one of the components in genus *Symphytum* L., has gastroprotective properties due to its anti-inflammatory properties [13]. According to other study, there is another component in genus *Symphytum* L. that contributes to the anti-inflammatory properties, which are choline and rosmarinic acid. Choline demonstrates its anti-inflammatory effect by activating alpha-7 nicotinic receptors and reducing cytokine production in macrophages. In the other hand, the rosmarinic acid prevents the synthesis of inflammatory mediators [42, 43]. In addition, several studies revealed that *Portulaca oleracea* L. had a variety of pharmacological actions, including anti-inflammatory properties [22]. Moreover, the protection and healing of both normal and damaged gastric mucosa are heavily dependent on blood flow. This is because blood flow supplies the mucosa with essential elements such as oxygen and HCO3 while also extracting harmful substances like H+ and toxic agents that leak from the lumen into the mucosa; thereby, offering effective protection, the hyperemic response enhances the delivery of HCO3- to the mucosal layer by fortifying the injured mucosa against inward diffusion of H+ and corrosive substances such as ethanol, offering adaptive protection, also gastric ulcers cause damage to blood vessels, but during the healing process, blood flow gradually returns to its normal rate. The healing of a gastric ulcer can be influenced by either stimulated or inhibited angiogenesis in the granulation tissue [44].

The current study suggests that genus *Symphytum* L. may have potential as a phytomedicine for treating peptic ulcers. However, further research is needed to evaluate the safety and efficacy of varying doses of genus *Symphytum* L. in human subjects before drawing definitive conclusions. Until comprehensive research has been conducted in human beings, a cautious approach is recommended when considering the use of genus *Symphytum* L. Standardized procedures for preparing herbal remedies are critical in ensuring consistent quality and purity. Additional research should be conducted using an expanded sample size and lengthier treatment durations to validate the findings of this study. Other research needs to study histological pathophysiology to evaluate plants' effects of genus *Symphytum* L. and *Portulaca oleracea* L. on aspirin-induced acute gastric ulcer in rats.

Despite the positive results shown by this study on the effects of genus *Symphytum* L. and *Portulaca oleracea* L. on gastric ulcers in rats, it is important to consider potential biases, such as experimental design variations including the specific dosage and method of administering aspirin to induce gastric ulcers for seven days, as well as the critical influence of sample size on the reliability and generalizability of the findings. Identifying and mitigating these biases is crucial for accurately assessing the efficacy of these plants and translating these results into potential human therapies.

## 5. Conclusions

In conclusion, the present study provides compelling evidence of the therapeutic properties of allantoin, kaempferol, and apigenin, particularly against agents that damage the gastric mucosa, such as nonsteroidal anti-inflammatory drugs (NSAIDs) including aspirin. The favorable impact of allantoin is linked with its antisecretory and cytoprotective pathways, possibly by increasing the levels of PGE2. These findings advance our understanding of the pharmacological mechanisms of these compounds and their potential therapeutic applications in managing gastric disorders. Accordingly, genus *Symphytum* L. *and Portulaca oleracea* L. could be used as medicinal plants as curative agents against gastric ulcer in experimental rats.

Financial support posed a significant challenge in this study as well as the quantity of rats, carrying out complicated scientific procedures such as isolating active compounds from plants and performing rigorous analyses and dissections on rats.

## Figures and Tables

**Table 1:** Effects of genus *Symphytum* L. and *Portulaca oleracea* L. on body weight gain in rats inflicted with gastric ulcer.

Groups		Aspirin and plant extracts	Doses (mg/kg b.wt.)	BWG (g/7 days)
				Mean ± SE
Control −ve	1	—	—	27.50 ± 0.09^a^
Control +ve	2	Aspirin (Asp)	200	10.00 ± 0.89^c^
Genus *Symphytum* L.	3	Genus *Symphytum* L. *+* (Asp)	100	16.60 ± 0.52^b^
*Portulaca oleracea* L.	4	*Portulaca oleracea* L. + (Asp)	100	10.90 ± 0.29^c^
L.S.D^∗^				1.05

Values denote arithmetic means ± standard error of the means. Means with different letters (a, b, c, and d) in the same column differ significantly at *p* ≤ 0.05 using the one-way ANOVA test, while those with similar letters are nonsignificant. ^∗^LSD: low significant difference.

**Table 2:** Effects of genus *Symphytum L.* and *Portulaca oleracea L.* on the length of gastric ulcer in rats inflicted with gastric ulcer.

Groups		Aspirin and plant extracts	Doses (mg/kg b.wt.)	Gastric ulcer length (mm)
				Mean ± SE
Control −ve	1	—	—	0.00
Control +ve	2	Aspirin (Asp)	200	2.03 ± 0.09^a^
Genus *Symphytum* L.	3	Genus *Symphytum* L. *+* (Asp)	100	0.91 ± 0.02^c^
*Portulaca oleracea* L.	4	*Portulaca oleracea* L. *+* (Asp)	100	1.21 ± 0.05^b^
L.S.D^∗^				0.12

Values denote arithmetic means ± standard error of the means. Means with different letters (a, b, c, and d) in the same column differ significantly at *p* ≤ 0.05 using the one-way ANOVA test, while those with similar letters are nonsignificant. ^∗^LSD: low significant difference.

**Table 3:** Effects of plant extracts on the volume of gastric juice in rats inflicted with gastric ulcer.

Groups		Aspirin and plant extracts	Doses (mg/kg b.wt.)	Volume of gastric juice (mL)
				Mean ± SE
Control −ve	1	—	—	1.33 ± 0.03^d^
Control +ve	2	Aspirin (Asp)	200	2.30 ± 0.08^a^
Genus *Symphytum* L.	3	Genus *Symphytum* L. *+* (Asp)	100	1.50 ± 0.04^c^
*Portulaca oleracea* L.	4	*Portulaca oleracea* L. *+* (Asp)	100	1.70 ± 0.07^b^
L.S.D^∗^				0.09

Values denote arithmetic means ± standard error of the means. Means with different letters (a, b, c, and d) in the same column differ significantly at *p* ≤ 0.05 using the one-way ANOVA test, while those with similar letters are nonsignificant. ^∗^LSD: low significant difference.

**Table 4:** Effects of genus *Symphytum L. Portulaca oleracea L.* on pH of gastric juice in rats inflicted with gastric ulcer.

Groups		Aspirin and plant extracts	Doses (mg/kg b.wt.)	pH of gastric juice
				Mean ± SE
Control −ve	1	—	—	4.70 ± 0.04^d^
Control +ve	2	Aspirin (Asp)	200	5.60 ± 0.09^ab^
Genus *Symphytum* L.	3	Genus *Symphytum* L. *+* (Asp)	100	5.50 ± 0.07^bc^
*Portulaca oleracea* L.	4	*Portulaca oleracea* L. *+* (Asp)	100	5.80 ± 0.08^a^
L.S.D^∗^				0.51

Values denote arithmetic means ± standard error of the means. Means with different letters (a, b, c, and d) in the same column differ significantly at *p* ≤ 0.05 using the one-way ANOVA test, while those with similar letters are nonsignificant. ^∗^LSD: low significant difference.

**Table 5:** Effects of genus *Symphytum L.* and *Portulaca oleracea L.* on the total acidity of gastric juice in rats inflicted with gastric ulcer.

Groups		Aspirin and plant extracts	Doses (mg/kg b.wt.)	Total acidity of gastric juice
				Mean ± SE
Control −ve	1	—	—	3.00 × 10^−2^ ± 0.001^d^
Control +ve	2	Aspirin (Asp)	200	1.00 × 10^−1^ ± 0.005^a^
Genus *Symphytum* L.	3	Genus *Symphytum* L. *+* (Asp)	100	3.50 × 10^−2^ ± 0.003^c^
*Portulaca oleracea* L.	4	*Portulaca oleracea* L. *+* (Asp)	100	5.00 × 10^−2^ ± 0.002^b^
L.S.D^∗^				0.001

Values denote arithmetic means ± standard error of the means. Means with different letters (a, b, c, and d) in the same column differ significantly at *p* ≤ 0.05 using the one-way ANOVA test, while those with similar letters are nonsignificant. ^∗^LSD: low significant difference.

**Table 6:** Effects of genus *Symphytum L.* and *Portulaca oleracea L*. on TG and TC in rats inflicted with gastric ulcer.

Groups		Aspirin and plant extracts	Doses (mg/kg b.wt.)	TG (mg/dl) mean ± SE	TC (mg/dl) mean ± SE
Control −ve	1	—	—	58.37 ± 1.03^d^	66.78 ± 0.99^d^
Control +ve	2	Aspirin (Asp)	200	98.92 ± 2.62^a^	130.30 ± 3.02^a^
Genus *Symphytum* L.	3	Genus *Symphytum* L. *+* (Asp)	100	80.24 ± 1.03^c^	78.24 ± 1.19^c^
*Portulaca oleracea* L.	4	*Portulaca oleracea* L. *+* (Asp)	100	95.15 ± 0.09^b^	103.31 ± 2.37^b^
L.S.D^∗^				3.05	9.19

Values denote arithmetic means ± standard error of the means. Means with different letters (a, b, c, and d) in the same column differ significantly at *p* ≤ 0.05 using the one-way ANOVA test, while those with similar letters are nonsignificant. ^∗^LSD: low significant difference.

**Table 7:** Effects of genus *Symphytum* L. and *Portulaca oleracea* L. on AST, ALT, and ALP in rats inflicted with gastric ulcer.

Groups		Aspirin and plant extracts	Doses (mg/kg b.wt.)	AST (U/L) mean ± SE	ALT (U/L) mean ± SE	ALP (U/L) mean ± SE
Control −ve	1	—	—	45.92 ± 2.01^d^	41.25 ± 1.17^d^	146.40 ± 4.07^d^
Control +ve	2	Aspirin (Asp)	200	130.13 ± 3.07^a^	116.40 ± 2.30^a^	292.90 ± 5.16^a^
Genus *Symphytum* L.	3	Genus *Symphytum* L. *+* (Asp)	100	80.49 ± 1.19^c^	59.99 ± 2.40^c^	191.90 ± 3.81^c^
*Portulaca oleracea* L.	4	*Portulaca oleracea* L. *+* (Asp)	100	93.01 ± 2.81^b^	71.18 ± 1.06^b^	212.20 ± 1.92^b^
L.S.D^∗^				5.51	3.08	6.01

Values denote arithmetic means ± standard error of the means. Means with different letters (a, b, c, and d) in the same column differ significantly at *p* ≤ 0.05 using the one-way ANOVA test, while those with similar letters are nonsignificant. ^∗^LSD: low significant difference.

**Table 8:** Effects of genus *Symphytum L.* and *Portulaca oleracea L*. on RBC, WBC, HGB, and PLT in rats inflicted with gastric ulcer.

Groups		Aspirin and plant extracts	Doses (mg/kg b.wt.)	RBC (10^6^/*μ*L) mean ± SE	WBC (10^3^/*μ*L) mean ± SE	HGB (g/dl) mean ± SE	PLT (10^3^/*μ*L) mean ± SE
Control −ve	1	—	—	8.13 ± 0.04^cd^	3.30 ± 0.02^d^	9.60 ± 0.07^d^	997.00 ± 5.33^d^
Control +ve	2	Aspirin (Asp)	200	9.62 ± 0.09^a^	6.11 ± 0.09^a^	14.88 ± 0.10^a^	1420.00 ± 9.91^a^
Genus *Symphytum* L.	3	Genus *Symphytum* L. *+* (Asp)	100	8.49 ± 0.03^bc^	4.47 ± 0.05^c^	12.20 ± 0.08 g/dl^c^	1142.00 ± 3.38^c^
*Portulaca oleracea* L.	4	*Portulaca oleracea* L. *+* (Asp)	100	8.84 ± 0.06^b^	5.13 ± 0.03^b^	14.60 ± 0.05 g/dl^ab^	1364.00 ± 7.20^b^
L.S.D^∗^				0.51	0.08	0.30	10.39

Values denote arithmetic means ± standard error of the means. Means with different letters (a, b, c, and d) in the same column differ significantly at *p* ≤ 0.05 using the one-way ANOVA test, while those with similar letters are nonsignificant. ^∗^LSD: low significant difference.

## Data Availability

All the data used to support the findings of this study are available within the article and no other data found at any elsewhere.
